# Brain lateralization and self-reported symptoms of ADHD in a population sample of adults: a dimensional approach

**DOI:** 10.3389/fpsyg.2015.01418

**Published:** 2015-09-22

**Authors:** Saleh M. H. Mohamed, Norbert A. Börger, Reint H. Geuze, Jaap J. van der Meere

**Affiliations:** ^1^Department of Clinical and Developmental Neuropsychology, Faculty of Behavioural and Social Sciences, University of GroningenGroningen, Netherlands; ^2^Department of Psychology, Beni-Suef UniversityBeni-Suef, Egypt

**Keywords:** self-report, adults population, ADHD symptoms, brain lateralization, dimensional approach

## Abstract

Many clinical studies reported a compromised brain lateralization in patients with Attention-Deficit/Hyperactivity Disorder (ADHD) without being conclusive about whether the deficit existed in the left or right hemisphere. It is well-recognized that studying ADHD dimensionally is more controlled for comorbid problems and medication effects, and provides more accurate assessment of the symptoms. Therefore, the present study applied the dimensional approach to test the relationship between brain lateralization and self-reported ADHD symptoms in a population sample. Eighty-five right-handed university students filled in the Conners’ Adult ADHD Rating Scales and performed a lateralization reaction time task. The task consists of two matching conditions: one condition requires nominal identification for letters tapping left hemisphere specialization (Letter Name-Identity condition) and the other one requires physical and visuospatial identification for shapes tapping right hemisphere specialization (Shape Physical-Identity condition). The letters or shapes to be matched are presented in left or right visual field of a fixation cross. For both task conditions, brain lateralization was indexed as the difference in mean reaction time between left and right visual field. Linear regression analyses, controlled for mood symptoms reported by a depression, anxiety, and stress scale, showed no relationship between the variables. These findings from a population sample of adults do not support the dimensionality of lateralized information processing deficit in ADHD symptomatology. However, group comparison analyses showed that subjects with high level of inattention symptoms close to or above the clinical cut-off had a reduced right hemisphere processing in the Shape Physical-Identity condition.

## Introduction

With a prevalence rate of 5% in children and 1 to 7% in adults (Polanczyk and Rohde, 2007; Polanczyk et al., 2007) Attention-Deficit/Hyperactivity Disorder (ADHD) is a common developmental disorder characterized by impaired levels of attention and/or hyperactive-impulsive behaviors. Apart from behavioral symptoms, subjects show various deficits in executive functions, response inhibition (Boonstra et al., 2005; Willcutt et al., 2005), and motivational functions (Metin et al., 2012, 2014).

Moreover, there is evidence that abnormal brain lateralization might be a core component underlying dysfunctions in ADHD (Hale et al., 2008, 2009). At the structural and neuroimaging level, studies have reported atypical right hemisphere structure (Valera et al., 2007; Frodl and Skokauskas, 2012); in particular, smaller size of right frontal and prefrontal cortex were found in subjects with ADHD (Hill et al., 2003; Almeida et al., 2010). Atypical right hemisphere structure may affect attentional processing and response inhibition (Stefanatos and Wasserstein, 2001; Hart et al., 2013). Furthermore, it may produce an increased rightward asymmetry for EEG alpha and beta waves (Swartwood et al., 2003; Hale et al., 2010; Jaworska et al., 2013).

Other studies have reported abnormalities in the left hemisphere; in particular, slightly greater left posterior cingulate cortex (Nakao et al., 2011) that relates to memory, emotions, and motivation by reward, and is involved in both the dorsal attentional network, and the fronto-parietal control network for executive motor control (Leech and Sharp, 2014). In addition, a smaller left caudate nucleus has been found in subjects with ADHD (Durston, 2003). This area contributes to the cognitive selection of actions schema and evaluates action-outcomes (Grahn et al., 2008).

At the behavioral level, some studies have suggested a disruption of the right hemisphere attentional network. For instance, studies using divided visual field tasks have indicated perceptual asymmetry deficit in ADHD characterized by poor performance for Left Visual Field (LVF) during visuospatial attentional processing (Carter et al., 1995; Sandson et al., 2000; Song and Hakoda, 2012). Self-reported inattention symptoms were related to less efficient orienting attention to the LVF (Poynter et al., 2010). Additionally, ADHD symptoms were positively correlated with the interference effects for Right Visual Field (RVF) targets under low perceptual load (Geeraerts et al., 2008; Chan et al., 2009). The severity of ADHD was also related to a higher proportion of errors for the left hemi-field on a visual scanning task (Braun et al., 2013) as well as on paper and pencil cancelation tests (Sandson et al., 2000; Jiang et al., 2008; Jones et al., 2008).

Other behavioral studies have suggested a reduced left hemisphere contribution during lexical decision (Hale et al., 2005) and dichotic listening tasks (Hale et al., 2006, 2008). The suggestion that the left hemisphere might be compromised in ADHD is also mirrored by the fact that subjects have difficulties in naming tasks, and it is well-recognized that the disorder has a considerable overlap with reading disorders (Tannock et al., 2000; Bellani et al., 2011; Levy et al., 2013; Tamm et al., 2014).

All in all, the information presented above on abnormal brain lateralization in ADHD is inconclusive; albeit most evidence favors right hemisphere dysfunction. In arriving at this conclusion, it is underlined that atypical laterality is based on research carried out on individuals fulfilling the DSM criteria for ADHD which is termed the categorical approach. The approach is criticized because many individuals who fulfill the diagnostic criteria for ADHD have more mental problems such as mood disorders, aggression, and learning disabilities. In addition, many of them use medication. The two factors of comorbidity and medication use may confound experimental data and interpretation. In the present study, we aim to investigate lateralized brain dysfunction at the behavioral level from the perspective of the dimensional approach. The dimensional approach does not require the arbitrary dichotomization of individuals into categories based on an all-or-none principle but positions individuals on a continuum (Parens and Johnston, 2009). It allows studying relationships between symptoms of ADHD and neuropsychological function or performance over a wide range of severity and in a wider population.

It is well-recognized that studying ADHD dimensionally is more controlled for comorbid problems and medication effects. By measuring comorbidities related to the disorder and including them in the analyses, researchers can evaluate the effect of ADHD and its comorbidities apart on the variables of interest. With respect to medication effects, the effects are supposed to be minor or not present if the majority of the sample reported no clinical diagnosis. Moreover, the dimensional approach usually offers a more powerful statistical test of any hypothesis due to the large sample size and also provides more accurate assessment of the symptoms (Hudziak et al., 2007). This may explain the increasing interest in studying ADHD as a quantitative trait rather than as a disorder. Much empirical support for studying ADHD as a continuum dimension are given by neuropsychological (Herrmann et al., 2009; Lubke et al., 2009; Jarrett et al., 2014), genetic (Nikolas and Burt, 2010; Larsson et al., 2012), and neuroimaging studies (Shaw et al., 2011; Hoogman et al., 2012). With the exception of Todd et al. (2001), researchers using a variety of statistical methods have concluded that ADHD in children, adolescents (Lubke et al., 2009), and adults (Marcus and Barry, 2011; Marcus et al., 2012) has a dimensional latent structure. Many of these dimensional studies explored the association between self-reported ADHD symptoms in adult population and both neurobiological variables, such as brain volume and cortical thickness, and laboratory measures of neuropsychological functioning, such as visual working memory tasks and the Stroop test.

The present study has investigated the dimensional relationship between brain lateralization and self-reported ADHD symptoms in a sample of adults. Especially, inattention symptoms are of interest here, as it has been found that adults demonstrate more inattention symptoms than other ADHD symptoms (for review see, Wilens et al., 2002; McGough and Barkley, 2004). To test brain lateralization at the behavioral/functional level, we used a visual reaction time task with verbal (letters) and nonverbal (shapes) stimuli applying the Banich (1998) design to measure brain lateralization. The design provides information about perceptual asymmetry as a function of hemispherical differences. The advantage of using Banich design is that it requires more attentional demands than other divided visual field designs and being most sensitive for ADHD difficulties in adults. One might question whether the Banich design is the most appropriate design for testing brain lateralization. Generally speaking, it is well recognized that there are possible confounders in measuring brain lateralization using divided visual field designs such as the Banich design (Bourne, 2006). One of the most critical issues related to the Banich design is whether it taps lateralization, a visual scanning bias, or both (Fecteau and Enns, 2005). The bias is defined in terms of reading habits; for example, left to right readers tend to perform better on LVF than on RVF trials (Nicholls and Roberts, 2002). In our task stimuli are presented diagonally, controlling for the potential effect of the scanning bias (Banich and Belger, 1990).

The original work of Banich and colleagues reported evidence that task performance is affected by lateralized brain functions: the right hemisphere functioning (Ladavas et al., 1984) and LVF performance are affected by sadness mood (Banich et al., 1992). We admit that the Banich task and its varieties were more often used to tap interhemispheric interaction compared to brain lateralization (Weissman and Banich, 1999; Banich et al., 2000; Compton, 2002; Passarotti et al., 2002; Lopez et al., 2007). Therefore, before addressing the main goal of the present study (self-reported ADHD symptomatology and its link with brain laterality), the validity of the Banich design to investigate lateralization has been tested using two different types of information processing (nominal versus orientation and physical processing).

The task consists of two matching conditions; the first, Letter Name-Identity condition, requires perceptual nominal identification for letters tapping left hemisphere specialization (Eviatar et al., 1994; Flowers et al., 2004). The second, Shape Physical-Identity condition, requires perceptual identification for the orientation and physical characteristics of shapes (nonverbal visuospatial processing) tapping right hemisphere specialization (Vogel et al., 2003). In the two task conditions, the matching letters or shapes are presented either within the LVF directly connected with the right hemisphere via neural pathways, or presented within the RVF with direct neural connection with the left hemisphere. The brain lateralization in perception is defined in terms of visual field advantage (faster reaction time on one visual field relative to the other). Within this framework, one can expect LVF/right hemisphere advantage in the shape matching condition and RVF/left hemisphere advantage in the letter matching condition. However, some reports indicated that LVF/right hemisphere advantage can be detected in tasks with rapid visual presentation such as the Banich task (Verleger et al., 2010) because right hemisphere contributes to perception and attentional processing when subjects are required to shift and focus attention (Corbetta et al., 1993; Nobre et al., 1997). We expect that LVF/right hemisphere advantage will be more pronounced in the shape matching condition rather than in the letter matching condition.

Most of the previous studies suggested that individuals with ADHD have a right hemisphere deficit; consequently, we hypothesize that a higher level of ADHD symptoms is related to a slower right hemisphere processing of perceptual information as indicated by a smaller size of LVF advantage, especially in the shape matching condition.

To measure self-reported ADHD symptoms, we used the Conners’ Adult ADHD Rating Scales (CAARS; Conners et al., 1999), a popular self-report containing the key domains of ADHD (inattention, hyperactivity, and impulsivity) and an “overall” index of ADHD symptomatology. The study takes depression, anxiety, and stress into account since it is well-known that these mood symptoms are common comorbidities related to ADHD symptomatology (Alexander and Harrison, 2013) and may affect the hemispheric functioning (see: Hecht, 2010). Thus, participants have to complete the Depression, Anxiety and Stress scale (DASS; Lovibond and Lovibond, 1995).

Briefly, the present study aims to answer the following questions:

(1) Do Letter Name-Identity and Shape Physical-Identity task conditions tap different sizes of visual field advantage measured by reaction time and error rate?
(2) After controlling for mood symptoms (depression, anxiety, and stress), do self-reported ADHD symptoms (inattention, hyperactivity, impulsivity, and ADHD index) predict the size of visual field advantage on each task condition?

## Materials and Methods

### Participants

Eighty-five first year psychology students (65 females) at the University of Groningen received course credit for their participation in the experiment. The exclusion criteria were: (a) reported former or current diagnosis with ADHD and/or a diagnosis concerning depression and/or anxiety/stress, (b) reported medication related to ADHD and/or mood disorders. The mean age was 20.3 years (*SD* = 2.4, min:max = 18:32). All participants were right handed; they were selected based on a criterion of having a score above 40 on the Edinburgh Handedness Inventory (Oldfield, 1971). Participants reported normal or corrected to normal vision.

### Questionnaires

Participants completed anonymously two self-reported questionnaires: the CAARS (Conners et al., 1999) and the DASS (Lovibond and Lovibond, 1995). The CAARS consists of 66 four-point items ranging from 0 (not all all) to 3 (very frequently), (e.g., “*I’m absent minded in daily life activity*”). The items are surveying four dimensions; three dimensions correspond to core features of ADHD (inattention/memory problems, impulsivity/emotional lability, and hyperactivity/restlessness). The fourth dimension corresponds to an important consequence of ADHD (i.e., problems with self-concept). The scale also contains the ADHD index subscale seen to be the most reliable and valid measure of overall ADHD symptomatology (Alexander and Harrison, 2013; Simon et al., 2013). In addition, the scale provides a built-in index to assess the response inconsistency; the index contains eight pairs of items that have similar content. The inconsistency index score is computed by summing the difference scores of each pair. A cutoff score of eight or greater indicates invalid responses. *T*-scores were derived to compare the individual’s responses to population norms. The scores were transformed from raw scores and have a mean of 50 and standard deviation of 10.

The DASS was used to estimate mood problems of the participants. The questionnaire is subdivided in three subscales: (a) depression, (b) anxiety, and (c) stress. Each subscale contains 14 items (e.g., an item for depression subscale “I felt sad and depressed”; for anxiety subscale “I felt terrified”; for stress subscale “I found it difficult to relax”). The participants rated how often each emotional state applied to them over the last week on a four-point scale ranging from 0 (did not apply to me at all) to 3 (applied to me very much). The sum score of each subscale classifies the participants into one of five categories (normal, mild, moderate, severe, and extremely severe) relative to the population mean.

The CAARS and DASS questionnaires have a high reliability and validity and are suited for the dimensional approach on psychopathology (Antony et al., 1998; Erhardt et al., 1999; Adler et al., 2008). The questionnaires have a high model-fit such that use of the American versions is justified to use across the globe (Christiansen et al., 2011; Ramli et al., 2012).

### Brain Lateralization Task

**FIGURE 1:**
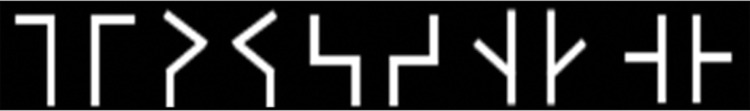
**Examples of match stimuli in the left and right visual field for the task conditions.** LVF = left visual field; RVF = right visual field.

The task was based on the Banich paradigm (Banich, 1998). The stimuli consist of a fixation cross and three letters or shapes arranged at the vertices of an invisible triangle. Two of the letters or shapes were presented above the fixation cross while the third was presented below (see **Figure 1**). The two letters or shapes above the fixation cross were the probes, and the letter or shape below the fixation cross was the target. One of the two probes had to be matched with the target. When a match is detected the subject has to press a response button.

**FIGURE 2:**
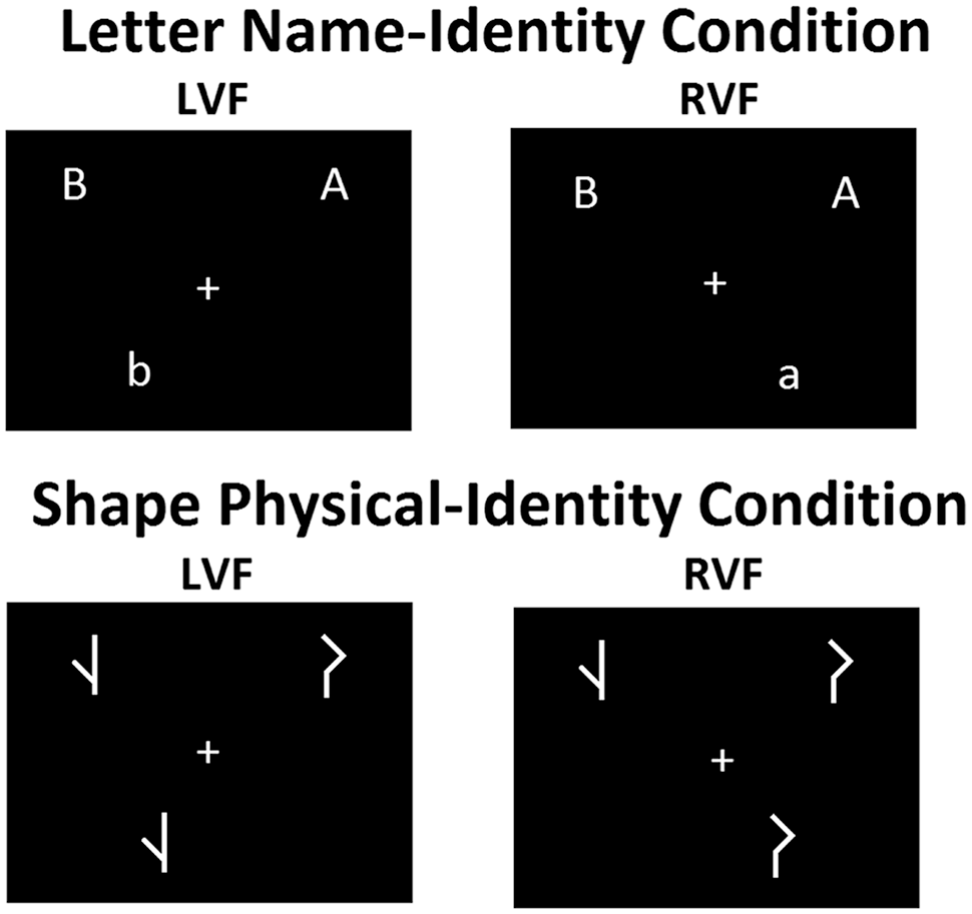
**Five shapes and their mirror shapes, each consists of three small connected lines of 2 mm long and line thickness of 2.25 point size**.

There were two matching conditions; in the first the Letter Name-Identity condition, letters were displayed in different cases (the probes in upper-case and the target in lower-case) and randomly chosen from the letters A, B, D, G, H, E, F, L, R, M, T, and Q. A match was defined when one of the probes and the target had same name identity regardless of the letter case. The match may rely on the phonetic code of the letter names tapping left hemisphere processing. In the second condition, Shape Physical-Identity, unfamiliar shapes (the probes as well as the target) were displayed in their original form or in their mirrored form (see **Figure 2**). A match was defined when one of the probes matched the target in the shape and orientation.

The total number of trials for each task condition was 80 trials. The match:mismatch ratio of the stimuli was 50:50. During a mismatch trial, no response was needed. Mismatch trials were included to prevent impulsive and careless responding. The presentation of stimuli was balanced over LVF and RVF. **Figure 1** presents examples of the match stimuli with matching letters or shapes presented in LVF or RVF. A trial started with a fixation cross for 1000 ms followed by a stimulus for 150 ms. Next, the fixation cross was presented for another 2000 ms, the trial ended with a black screen for 500 ms.

The probes were presented 1.6° above the fixation cross while one probe was presented 2.68° to the left and the other 2.68° to the right of the fixation cross. The target was presented 1.6° below and 1.6° to the left or the right of the fixation cross. The fixation cross was located in the center of the screen. All letters and shapes had the same dimensions of 0.95° horizontally and 1.3° vertically. Stimuli were presented in white color on a black background to reduce the light emitted from LED screen.

#### Apparatus

The task was conducted on a laptop computer using E-Prime software version 2.0 to control the stimulus presentation and to specify the correct and incorrect responses. The visual stimuli were displayed on a LED-backlit HD anti-glare screen with 1024 × 768 pixel resolution and a refresh rate of 60 Hz. A chin rest was used to fix the distance (50 cm) between the screen and participant’s eye. A response box of one button was used to record the reaction time. It was positioned half way between the chin rest and the screen to enable easy reach.

### Procedures

The study procedures were approved by the ethical committee governing psychology at the University of Groningen. Before running the experiment, the examiner explained the study procedures to the participants and obtained written informed consent. Thereafter, participants filled in the questionnaires and performed the brain lateralization task in counterbalance order (questionnaires-brain lateralization task/brain lateralization task-questionnaires). To perform the task, the participants were seated in a dimly lit room, their chin upon the chin rest. They were instructed to press a button with their right hand as fast and accurate as possible when the target letter or shape matches one of two probe letters or shapes. It was emphasized to keep their gaze on the fixation cross all the time and not to move their eyes away when the stimuli appeared. For eye blinks, the participants were verbally informed to make their possible blinks directly after pressing the button. This procedure aimed to decrease the number of missing errors caused by eye blinking. We admit that using instruction to control eye movements is not the most effective method. It could be recommended to use an objective measure to monitor eye movements such as eye-tacking or electrooculography method. However, using such experimental equipment is time consuming in terms of experimental preparation, and is less flexible in terms of the participants. We decided to control for eye movements by using the most often used method in the literature: instructing the participant to fixate on a central point together with rapid lateralized stimulus presentation of 150 ms, which is faster than the latency of eye movements (generally about 200 ms and even longer in subjects with ADHD: Munoz et al., 2003). As a result, an anticipated eye movement will lead to missing the stimulus and will be counted as an error.

Before each task condition, participants performed practice trials until they met a criterion of seven correct responses in any consecutive 10 trials. After reaching this criterion, the practice trials automatically terminated.

### Data Analysis

Reaction times of correct match trials and error rate in LVF and RVF were recorded. Error rate was calculated as the number of no response for match trials divided by the number of match trials to test performance consistency and possible speed–accuracy trade-off.

For reaction time and error rate performance, the crucial index of brain lateralization is calculated (in terms of visual field advantage) in each task condition as the relative difference between RVF and LVF performance: [(RVF–LVF)/(overall mean of within visual field trials)] × 100. Consequently, a compromised right hemisphere processing is reflected by longer LVF reaction time giving a small size of visual field advantage, especially in the shape matching condition; whereas, a compromised left hemisphere processing is reflected by a high value of brain lateralization index especially in the letter matching condition.

We run two statistical analyses:

(1) To test the difference between the two task conditions in brain lateralization, a repeated measure analysis of variance on the size of visual field advantage was performed. The within subject factor was task condition (Letter Name-Identity and Shape Physical-Identity). To test performance consistency and possible speed-accuracy trade-off, the size of visual field advantage was calculated separately from mean reaction time and error rate.
(2) The score on the ADHD index subscale of the CAARS is seen to be the most reliable measure of overall ADHD symptomatology. To test whether overall ADHD symptomatology can predict atypical brain lateralization, a linear regression analysis on the size of visual field advantage was performed using the ADHD index score as a predictor.

To test whether mood symptoms confound the effects of ADHD symptoms on brain lateralization, the mood DASS subscales were included in the analysis using a backward elimination procedure. In the first step of the procedure, the effects of all mood subscales combined (anxiety, depression, and stress) are tested followed by deleting, one by one, the mood subscales that are least significant.

The scores on the inattention, impulsivity, and hyperactivity subscales of the CAARS are reflecting the three key domains of ADHD symptoms. To test which specific symptoms (domain) can predict brain lateralization, a second linear regression analysis on the size of visual field advantage was performed using the scores on the inattention, impulsivity, and hyperactivity as predictors. The effects of the mood symptoms are tested in the same manner as in the first regression analysis using a backward elimination procedure. *Please note: the design of the task mainly relies on reaction time performance; therefore, visual field advantage was calculated using only reaction times, not the error rates.*

Finally, in subsequent analyses, we thought to confirm and test whether the relationship between lateralization and ADHD symptoms is present at the categorical not the dimensional perspective. Repeated measures analyses of variance were performed to compare the size of visual field advantage of first and fourth quartile group on the ADHD index, inattention, hyperactivity, and impulsivity scores. The within subject factor was task condition (Letter Name-Identity and Shape Physical-Identity) and the between subjects factor was group (Low-score, High-score).

## Results

### Questionnaires

**FIGURE 3:**
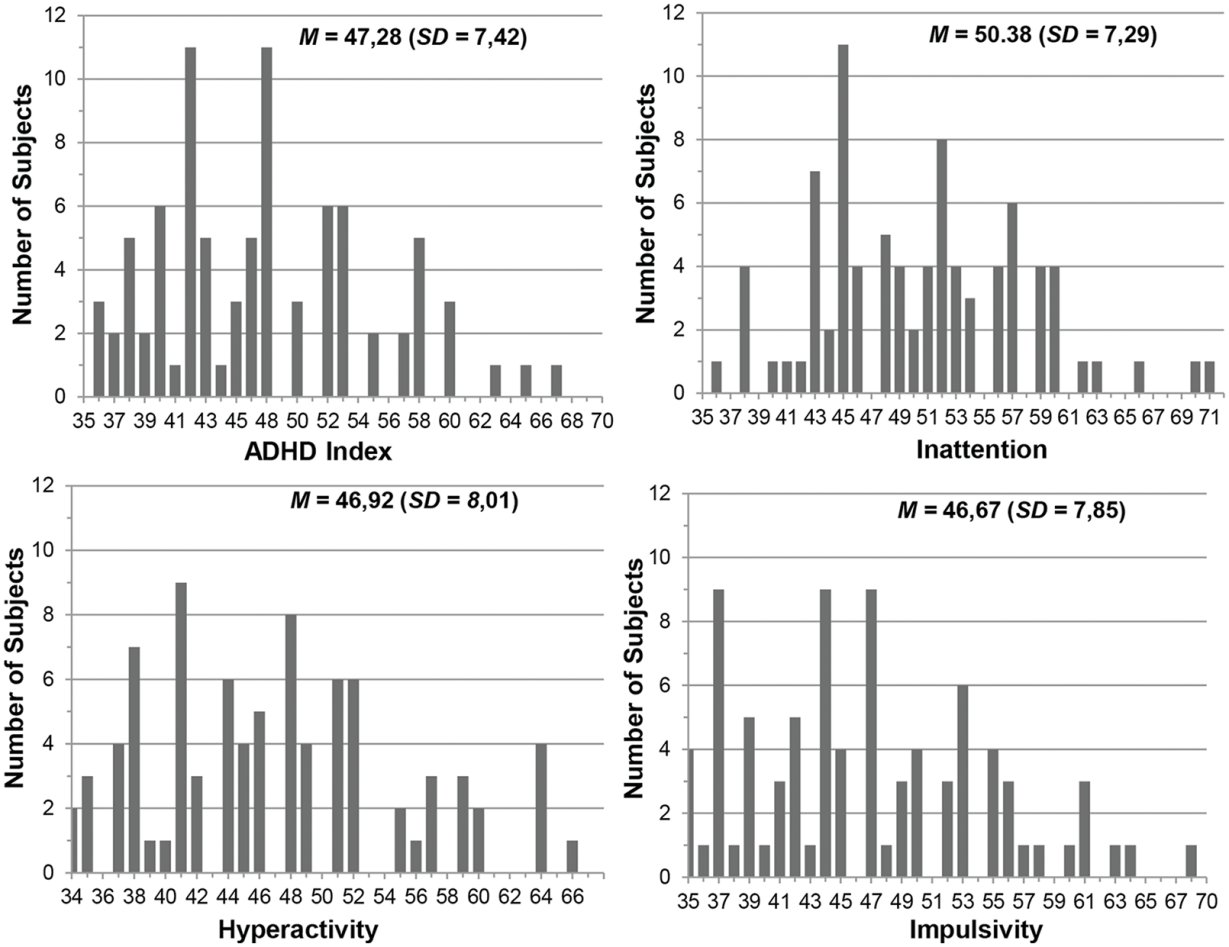
**The distribution of *T*-scores on the Conners’ Adult ADHD Rating Scales (CAARS) subscales of inattention, hyperactivity, impulsivity, and Attention-Deficit/Hyperactivity Disorder (ADHD) index**.

**Figure 3** shows the distribution of *T*-scores on the ADHD index, inattention, hyperactivity, and impulsivity subscales of the CAARS measuring the overall ADHD symptoms and its key domains. The figure indicates that scores on the four domains provide enough variance to test our lateralization hypothesis using the dimensional approach. According to the CAARS manual, the *T*-score of 65 can be used as a clinical cut-off for all subscales of the CAARS. As can be seen, few students scored above the clinical cut-off. Participants had reliable responses on the CAARS indicated by lower score than eight on the inconsistency index. **Table 1** presents the number of subjects within the cut-off scores for the DASS reflecting the degree of severity of mood symptoms relative to the population.

**Table 1:** Number of subjects scoring in various ranges on each subscale of the Depression, Anxiety and Stress scale (DASS).

DASS Subscale	Normal	Mild	Moderate	Severe	Extremely severe
Depression	73	9	2	1	0
Anxiety	63	8	10	3	1
Stress	64	7	13	1	0

Taken together, **Figure 3** and **Table 1** indicate that the sample can be seen as a population sample. Correlations between the overall ADHD symptoms (ADHD index subscale) and mood symptoms (DASS subscales) ranged from 0.31 to 0.43 with *p* < 0.005. This indicates that the closer the ADHD symptomatology comes to the clinical cut-off, the more pronounced the mood comorbidities.

### Brain Lateralization Task

#### Brain Lateralization Calculated from Reaction Time

**FIGURE 4:**
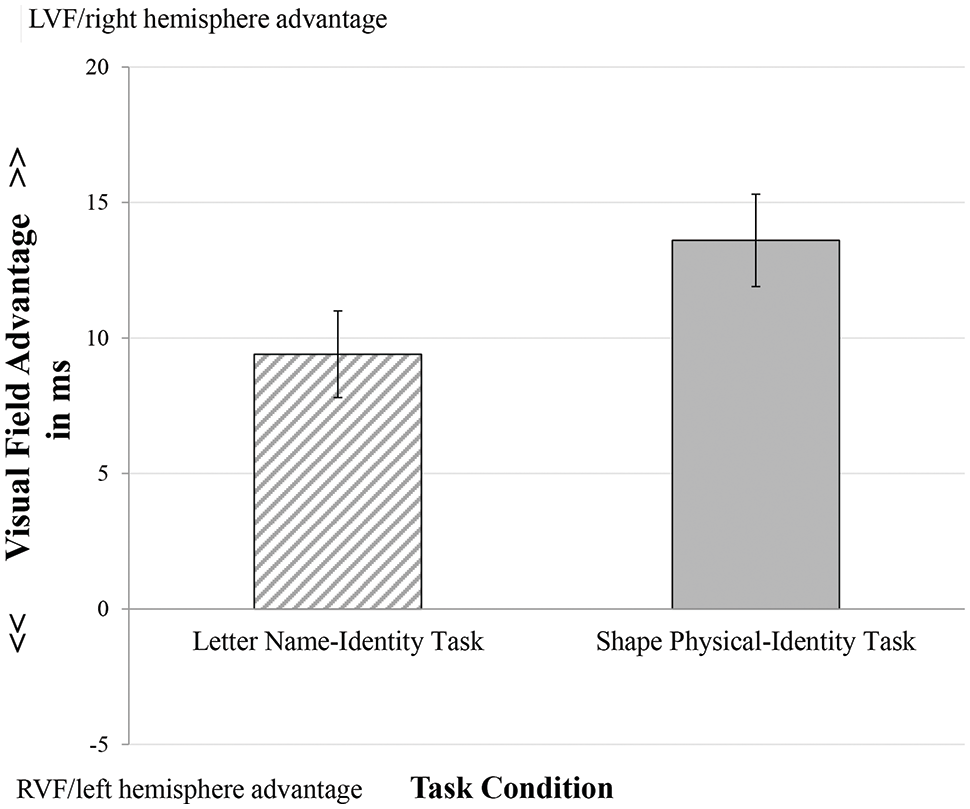
**Mean visual field advantage measured by reaction time for the task conditions.** Error bars indicate standard errors.

Neither the main effect of gender nor its interaction with the task condition was significant for the visual field advantage on reaction time (*p* ≥ 0.33). Therefore, **Figure 4** presents the mean size of visual field advantage for each task condition in males and females together.

A repeated measure analysis of variance revealed a significant main effect of task condition for the visual field advantage, *F*(1,84) = 4.029, *p* < 0.05, $\eta_{p}^{2}$ = 0.046, indicating that task conditions differ in visual field advantage. The Shape Physical-Identity condition had a higher LVF/right hemisphere advantage (*M* = 13.66, *SD* = 15.72) than the Letter Name-Identity condition (*M* = 9.49, *SD* = 15.12).

#### Brain Lateralization Calculated from Error Rate

The main effect of gender and its interaction with the task condition was not significant for the visual field advantage on error rate (*p* ≥ 0.42). The analysis revealed a non-significant main effect of task condition, *F*(1,84) = 3.424, *p* = 0.07, $\eta_{p}^{2}$ = 0.039. The Letter Name-Identity condition had a similar visual field advantage to the Shape Physical-Identity condition. The visual field advantage in the Shape Physical-Identity condition was close to zero.

The results indicated no speed-accuracy trade-off when we consider higher order processing reflecting a consistent task performance to test brain lateralization.

### The Relation between Brain Lateralization and Self-Reported ADHD and Mood Symptoms

**Table 2** shows the final models of the regression analyses in both task conditions. The analyses indicated that neither the ADHD index, nor the three key domains of ADHD (Inattention, Hyperactivity, and Impulsivity subscales of the CAARS) predict the visual field advantage calculated form reaction time performance on the task conditions (*R*^2^ ≤ 0.06, *p* ≥ 0.11). Pearson correlations between the size of visual field advantage and the CAARS subscales were not significant (*p* ≥ 0.16). In addition, the analyses showed that the DASS mood subscales did not relate to the size of visual field advantage. Therefore, it might be concluded that mood symptoms did not confound the outcome.

**Table 2:** The models of regression analyses predicting the size of visual field advantage from scores on the Attention-Deficit/Hyperactivity Disorder (ADHD) index, inattention, hyperactivity, and impulsivity subscales.

Predictors	Letter Name-Identity					Shape Physical-Identity				
	Coefficients		Model			Coefficients		Model		
	β	*T*	*R*	*R*^2^	Adjusted *R^2^*	β	*T*	*R*	*R*^2^	Adjusted *R*^2^
ADHD Index	–0.06	–0.51	0.06	0.00	–0.01	–0.02	–0.16	0.02	0.00	–0.01
Inattention	–0.16	–1.36	0.19	0.04	0.00	–0.20	–10.71	0.25	0.06	0.03
Hyperactivity	–0.12	–0.97				–0.14	–1.10			
Impulsivity	0.14	1.10				0.23	1.78			

*The models were non-significant. Because the DASS mood subscales did not affect or contribute to the variance of visual field advantage, they were removed from the presented models in the table.*

**FIGURE 5:**
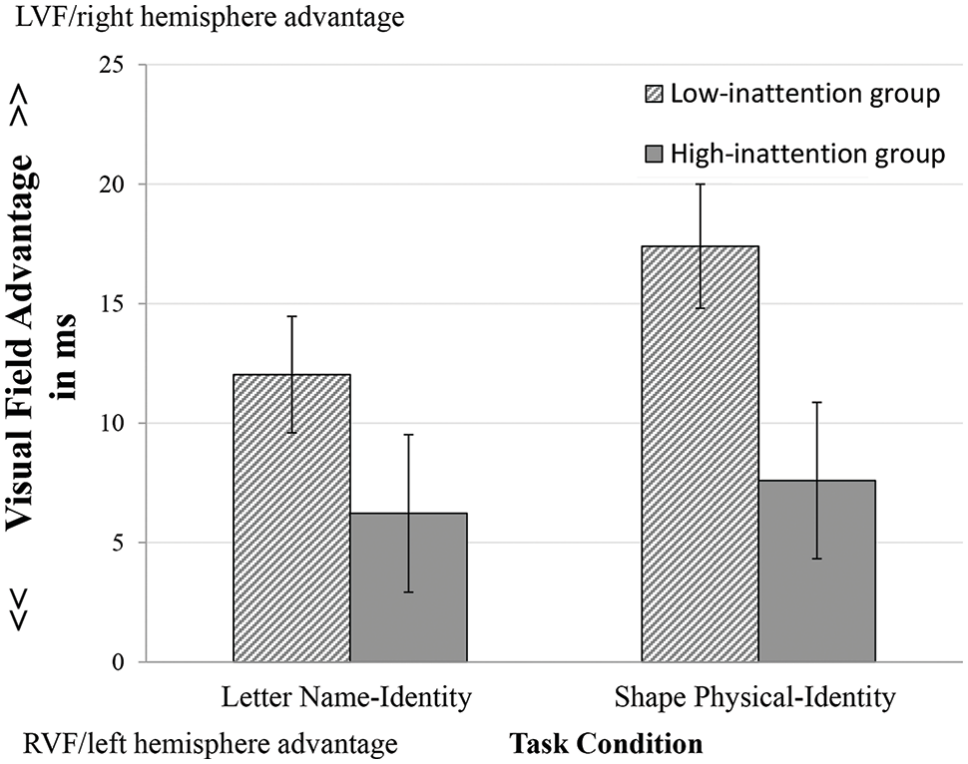
**Visual field advantage in the groups with High- and Low-score on inattention subscale.** Error bars indicate standard errors.

Comparing participants in the first and fourth quartile scores on inattention, hyperactivity and impulsivity yielded a significant difference in the size of visual filed advantage for group composition based on inattention. The High-score group (*n* = 23) on the inattention subscale showed a significant lower LVF/right hemisphere advantage than the Low-score group (*n* = 28); the main effect of group was significant *F*(1,49) = 5.97, *p* < 0.02, $\eta_{p}^{2}$ = 0.11. *Post hoc* analysis for the inattention subscale indicated that group differences were in the Shape Physical-Identity condition, *t*(49) = 2.38, *p* = 0.02, but not in the Letter Name-Identity condition, the mean size of visual field advantage were 6.27 and 12.03 in the Letter Name-Identity condition, and were 7.56 and 17.40 in the Shape Physical-Identity condition for, respectively, the High- and the Low-score group (see **Figure 5**). Groups did not differ on the other scales in the two task conditions.

## Discussion

The main conclusion of the present study is that there is no evidence for the dimensionality of atypical lateralized processing in ADHD symptomatology. However, at the level of group differences atypical lateralization with poor right hemisphere processing was linked to self-reported inattention symptoms.

Before discussing the study goal, we will discuss the validity of the Banich design to test brain lateralization. First, in line with the laterality literature showing LVF advantage in attentional orientation (Vogel et al., 2003; Asanowicz et al., 2012), higher size of LVF advantage was found in the shape matching condition than in the letter condition, suggesting more involvement of the right hemisphere processing in orientation and physical identification. It might be argued that positive scores of visual field advantage in the letter matching condition may reflect a visual scanning bias from the left to right side of the screen causing faster performance on LVF compared to RVF trials. If it is the case, the effect of the scanning bias should be present (balanced) in both task conditions. Put in other words, the scanning bias did not confound the laterality effects in comparing the task conditions. An alternative explanation is that rapid visual presentation, shifting and focused attention in our task may lead to right hemisphere dominance (Corbetta et al., 1993; Nobre et al., 1997; Verleger et al., 2010) which, in turn, produce LVF advantage in both task conditions.

Second, it is argued that the Banich design requires more attentional demands than other divided visual field designs (Banich and Belger, 1990; Bourne, 2006) and, as a result, being most sensitive for ADHD difficulties in adults. Therefore, the finding that self-reported inattention symptoms are related to lateralized performance (at least as far as the two extremes on the inattention dimension are concerned) might be seen as a validation of the Banich design to measure lateralized attentional processing. In sum, our task is valid to test at least the right hemisphere hypothesis in ADHD symptomatology.

At the behavioral level, no dimensional association between lateralization and ADHD symptoms was found. However, at the neuroimaging level atypical lateralized brain activity has been found in many studies during rest, simple or complex task performance (Sieg et al., 1995; Chabot and Serfontein, 1996; Ernst et al., 1998; Baving et al., 1999; Tamm et al., 2006; Hale et al., 2009; Cortese et al., 2012; Cubillo et al., 2012). This might suggest that atypical laterality is present in ADHD, but had not reached a specific degree or threshold to affect dimensionally the behavioral performance (e.g., reaction time). The threshold might be reached if ADHD scores are close to the clinical cut-off (65 or higher). Evidence in favor of this suggestion comes from a groupwise analysis between participants with low and high scores on the inattention subscale (the first versus the fourth quartile scores), the analysis revealed less LVF advantage in the shape matching condition for subjects with high inattention symptoms.

The result of group comparison analyses (categorical approach) is consistent with the clinical studies on children and adults with ADHD showing a compromised reaction time performance on the LVF (Carter et al., 1995; Epstein et al., 1997; Lubow et al., 2005; Geeraerts et al., 2008; Chan et al., 2009). Consequently, atypical lateralization at the behavioral level might be a characteristic of clinical ADHD as far as inattention type is concerned. Arrived at this point it is of interest to mention that the present study has a similar outcome as the Poynter et al. (2010) study. In their study, the CAARS was completed by university students. A categorization between groups with low and high scores on the inattention subscale showed that the group with high scores had less efficient orientation attention to the LVF during an attention network task. Inattention symptoms might be related to right hemisphere dysfunction, as it has been found that the right hemisphere is dominant in attentional processing (Shulman et al., 2010; Corbetta and Shulman, 2011; de Schotten et al., 2011).

The main conclusion that high level of inattention symptoms is associated with reduced right hemisphere function will be discussed in the view of some essential issues connected with the experimental design used in the present study.

First, the effect of lateralized motor components/processes on task performance was not counted because the study was not designed to explore lateralized motor processes. The study was designed to explore lateralized perceptual processes in the letter and the shape matching condition, and its relation to ADHD symptoms. To address the lateralized perceptual processes, the main manipulation of the task was focused on stimuli type (letters vs. oriented shapes) and using two visual fields (LVF vs. RVF). We admit that nevertheless a motor component was involved in the task, namely in the LVF condition. Consequently, it is questioned whether less LVF advantage as found in our study mirrors reduced right hemisphere processing, or abnormal interhemispheric communication in individuals with high inattention scores. Following Poffenberger’s logic, if the right hand is the responding hand then reaction time in the LVF should be slower than reaction time in the RVF because the time needed to transfer information between the two hemispheres is added to the total processing duration (Chaumillon et al., 2014). Our study findings showed the opposite. Therefore, it is likely that the LVF condition in our study reflected right hemisphere processing. A further support to the conclusion that less LVF advantage reflected reduced right hemisphere processing is that group differences were only found in the shape matching condition tapping right hemisphere specialization. This conclusion is in concert with many findings in the literature indicating a right hemisphere dysfunction together with intact or even faster interhemispheric interaction in individuals with ADHD (Carter et al., 1995; Sandson et al., 2000; Stefanatos and Wasserstein, 2001; Brown and Vickers, 2004; Rolfe et al., 2007; Song and Hakoda, 2012; Mohamed et al., 2015).

Second, although the outcome of the present study suggests that right hemisphere dysfunction and not compromised interhemispheric interaction is at stake in individuals with high inattention symptoms, it must be underlined that evidence is growing that there is a dynamic relation between lateralized brain functions and interhemispheric interaction (Serrien et al., 2006; Doron et al., 2012), and that especially the dynamic relation between right hemisphere functioning and interhemispheric interaction might be compromised in ADHD (Hale et al., 2008).

So far, we discussed brain lateralization in terms of functional asymmetry. The question emerges to what extent the functional asymmetry is related to anatomical asymmetry. In healthy subjects the LVF advantage, reported in several visuospatial tasks such as covert attention task, has been associated with a bilateral network including dorsal and ventral fronto-partial attention related systems and subcortical structures, i.e., thalamus, basal ganglia, and brainstem (Kastner and Ungerleider, 2000; Lawrence et al., 2003; Siman-Tov et al., 2007). Siman-Tov et al. (2007) discussed that the observed LVF advantage in healthy subjects may rely on the connectivity within the right hemisphere and/ or from the right to the left hemisphere. In individuals with attention deficit disorder poor attention to the LVF has been connected to white matter abnormalities, and disturbed interhemispheric connectivity (Castellanos et al., 2002; Roessner et al., 2004; Ashtari et al., 2005). In a review by Stefanatos and Wasserstein (2001), the authors reported that differential involvement of the right hemisphere in attention systems may provide a pathophysiological basis for differentiated subtypes of ADHD and that inattentive subtype is related primarily to right posterior involvement that is associated with impaired spatial processing causing nonverbal learning disabilities.

The dorsal and ventral attentional networks form key component of attentional regulatory systems of the brain. The former is closely related to circuits refer to as “salience network” and interrupting on going activity when appropriate. The network most likely to be affected by the ventral is the dorsal attentional network which mediates goal-directed, top–down executive control processes. The specificity of our task conditions may have challenged the ventral and dorsal networks. So far, most of the literature does not support clear involvement in the ventral attentional network in ADHD (Vossel et al., 2014), but some studies suggested a compromised ventral network (Janssen et al., 2015). It has been proposed that the ventral system is lateralized to the right hemisphere of the brain (Corbetta et al., 2008). Consequently, our present finding that reduced processing in the right hemisphere is associated with high inattention symptoms is not incompatible with the compromised ventral attentional system in ADHD. However, the dorsal network is supposed to be organized bilaterally. Hence, the reduced processing in the right hemisphere might also be due to the compromised dorsal network, and/or its interplay with the ventral network. These questions call for future research using the Banich paradigm in tandem with fMRI.

It is obvious that translating functional asymmetry into anatomical asymmetry needs the combination of a valid behavioral task and using fMRI. We hope that the present study provided some guidelines for the set-up of essential studies on ADHD symptomatology and brain laterality.

The final considerations concerns the characteristics of the participating sample. Although a sample of university students may be considered more homogenous than clinical samples with respect to IQ level, demographic variables, and comorbidities related to ADHD, the sample may still have hidden disabilities such as learning and psychiatric disorders (Wolf, 2001). We admit that we did not control for these hidden comorbidities, instead we controlled for the common mood comorbidities. Hale et al. (2010) underlined that abnormal brain lateralization is a common inherent feature of many psychiatric disorders. According to the authors, it is a challenge to find out whether disorders show different patterns of abnormal lateralization. Consequently, it may be possible that our outcomes are confounded by hidden disabilities.

Another factor that may have confounded the outcome is gender; our sample has more females than males. Meta-analytic reviews indicated that the prevalence rate of ADHD is higher in males than in females, and that there are gender differences in cognitive impairments, type of ADHD-comorbidities (Gershon and Gershon, 2002; Simon et al., 2009), and lateralized brain functions (Kret and De Gelder, 2012; Tomasi and Volkow, 2012; Herlitz and Lovén, 2013). Although the CAARS scores are corrected for gender and the reaction time outcomes showed no difference between males and females, the present findings need a replication examining effects of gender in a sample with more equal gender distribution.

It is well-recognized that the validity of self-reports from students may be questioned. For instance, students may understate or exaggerate rating themselves as having significant clinical ADHD symptoms (Harrison et al., 2007). Please note that in our sample the majority responded in a reliable way as estimated by the score on inconsistency index of the CAARS.

The present study focused on right handed adults, it could be of interest to investigate the same relationship on ambidextrous or left handed adults. Since ADHD symptoms are related to non-right handedness (Goez and Zelnik, 2008; Rodriguez et al., 2010), the detection of abnormal brain lateralization in ADHD may be more pronounced in ambidextrous or left handers.

In sum, we adopted the strategy of investigating the relevance of atypical lateralization for ADHD by using the dimensional approach. We assume that it provides more accurate assessment of the disorder than clinical classification. The results do not support, in a strict sense, the dimensionality of abnormal lateralized information processing in adult ADHD symptomatology, but underlines the role of atypical lateralization (right hemisphere deficit) in especially the inattention subtype of ADHD.

## Conflict of Interest Statement

The authors declare that the research was conducted in the absence of any commercial or financial relationships that could be construed as a potential conflict of interest.
